# Supplementary material for: Splice-Junction-Based Mapping of Alternative Isoforms in the Human Proteome
Source: Cell Rep. Author manuscript; Available in PMC 2020 Jan 15. (PMC6961840; doi:10.1016/j.celrep.2019.11.026)
Supplement: 2 [file NIHMS1546469-supplement-2.zip › DF1/PXD009737/TestisLysC/TestisLysC_6_ODF2_VLRAPCGAPSVTVTKPTMK.pdf]

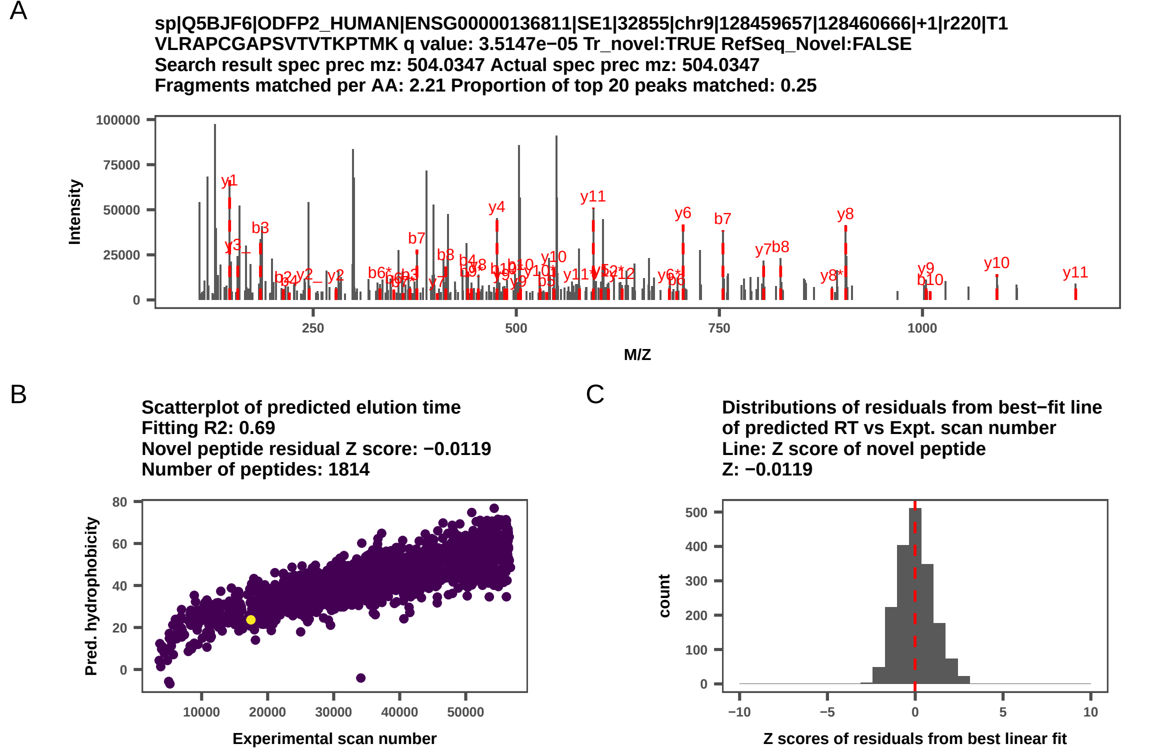
A
sp|Q5BJF6|ODFP2_HUMAN|ENSG00000136811|SE1|32855|chr9|128459657|128460666|+1|r220|T1
VLRAPCGAPSVTVTKPTMK q value: 3.5147e−05 Tr_novel:TRUE RefSeq_Novel:FALSE
Search result spec prec mz: 504.0347 Actual spec prec mz: 504.0347
Fragments matched per AA: 2.21 Proportion of top 20 peaks matched: 0.25
Intensity
100000
75000
50000
25000
0
250
500
750
1000
M/Z
y1
b3
y3
b7
b8
y4
y11
y10
y6
b7
y7
b8
y8
y9
b10
y10
y11
B
Scatterplot of predicted elution time
Fitting R2: 0.69
Novel peptide residual Z score: −0.0119
Number of peptides: 1814
Pred. hydrophobicity
80
60
40
20
0
10000
20000
30000
40000
50000
Experimental scan number
C
Distributions of residuals from best−fit line
of predicted RT vs Expt. scan number
Line: Z score of novel peptide
Z: −0.0119
count
500
400
300
200
100
0
−10
−5
0
5
10
Z scores of residuals from best linear fit
